# Case Report: Collapsing Focal Segmental Glomerulosclerosis After Initiation of Ado-Trastuzumab Emtansine Therapy

**DOI:** 10.3389/fonc.2021.796223

**Published:** 2021-11-29

**Authors:** Samy Hakroush, Svenja Wulf, Julia Gallwas, Björn Tampe

**Affiliations:** ^1^ Institute of Pathology, University Medical Center Göttingen, Göttingen, Germany; ^2^ Department of Gynecology and Obstetrics, University Medical Center Göttingen, Göttingen, Germany; ^3^ Department of Nephrology and Rheumatology, University Medical Center Göttingen, Göttingen, Germany

**Keywords:** focal segmental glomerulosclerosis, collapsing FSGS, tubular injury, ado-trastuzumab emtansine, T-DM1, acute kidney injury, proteinuria, nephrotic syndrome

## Abstract

Ado-trastuzumab emtansine (T-DM1) is an antibody–drug conjugate consisting of the monoclonal antibody trastuzumab linked to the maytansinoid DM1 with potential antineoplastic activity and is approved for human epidermal growth factor receptor 2 (HER2)-positive breast cancer. An analysis of the US Food and Drug Administration (FDA) Adverse Event Reporting System identified 124/1,243 (10%) renal adverse events for trastuzumab. However, there are no published case reports describing kidney biopsy findings related to nephrotoxicity of either trastuzumab or T-DM1. We report kidney biopsy findings in a case of nephrotic range proteinuria due to collapsing focal segmental glomerulosclerosis (FSGS) and tubular injury after initiation of T-DM1 therapy. After systematic exclusion of other causes, it is likely that the observed collapsing FSGS was associated with the prior initiation of T-DM1 therapy. This is further supported by the clinical course with improvement of proteinuria and kidney function 3 weeks after discontinuation of T-DM1 therapy without further specific treatment. In summary, we provide the first report of kidney biopsy findings in a case of nephrotic range proteinuria after initiation of T-DM1 therapy due to collapsing FSGS. This issue is especially relevant since T-DM1 is widely used, and nephrologists have to be aware of this potentially rare but severe complication.

## Introduction

The association between kidney disease and cancer has long been recognized but has only recently received full attention as a nephrological subspecialty, called onconephrology (1). Cancer patients may develop a variety of kidney lesions that not only impair their immediate survival but also limit the adequate treatment of the underlying malignant disease. Identifying novel mediators that regulate the growth and death of cancer cells has facilitated the development of more effective anticancer drugs that have revolutionized treatment options and clinical outcomes in cancer patients. However, many of these new drugs are often accompanied by significant side effects, including kidney injury (2). Ado-trastuzumab emtansine (T-DM1) is an antibody–drug conjugate consisting of the monoclonal antibody trastuzumab linked to the maytansinoid DM1 *via* a non-reducible thioether linkage with potential antineoplastic activity. The trastuzumab moiety of this conjugate binds to human epidermal growth factor receptor 2 (HER2) on the tumor cell surface and is subsequently internalized, therefore releasing the DM1 moiety to bind to tubulin with disruption of microtubule assembly/disassembly dynamics and inhibiting cell division and the proliferation of cancer cells that overexpress HER2 (3). T-DM1 has been approved specifically for pretreated metastatic HER2-positive breast cancer patients or for breast cancer patients with HER2-positive unresectable locally advanced or metastatic breast cancer who had previously received trastuzumab and taxanes (4). Moreover, T-DM1 has been approved for extended use to the adjuvant therapy of adult patients with HER2-positive early breast cancer who have residual invasive disease in the breast and/or lymph nodes after neoadjuvant taxane-based and HER2-targeted therapy (5). An analysis of the US Food and Drug Administration (FDA) Adverse Event Reporting System revealed that renal adverse events are observed in a considerable subset of patients treated with trastuzumab (6). However, there are no published reports describing kidney biopsy findings related to nephrotoxicity of either trastuzumab or T-DM1. We provide report kidney biopsy findings in a case presenting with deterioration of kidney function and nephrotic range proteinuria due to collapsing focal segmental glomerulosclerosis (FSGS) after initiation of T-DM1 therapy.

## Case Description

**Figure 1:**
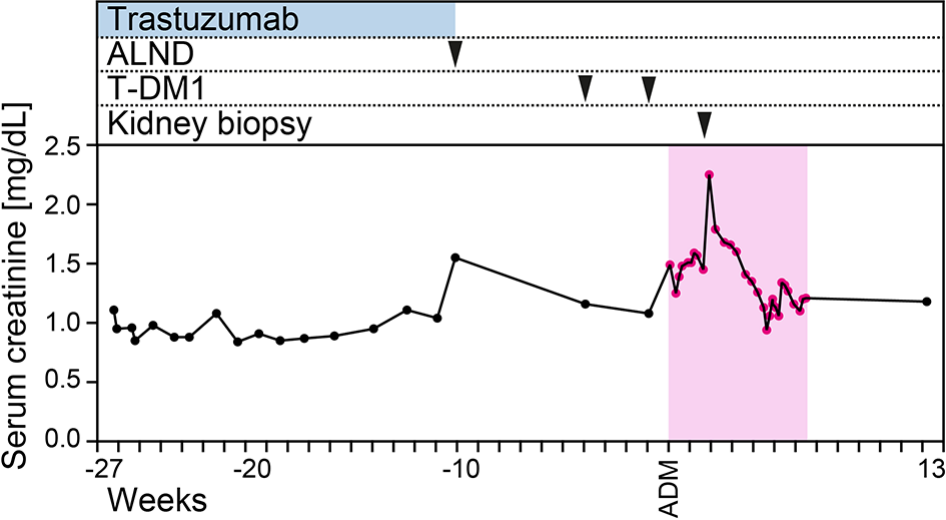
Time course of the case. Time course of serum creatinine levels, trastuzumab and T-DM1 infusions, and kidney biopsy. Abbreviations: ADM, admission; ALND, axillary lymph node dissection; T-DM1, ado-trastuzumab emtansine.

**Figure 2:**
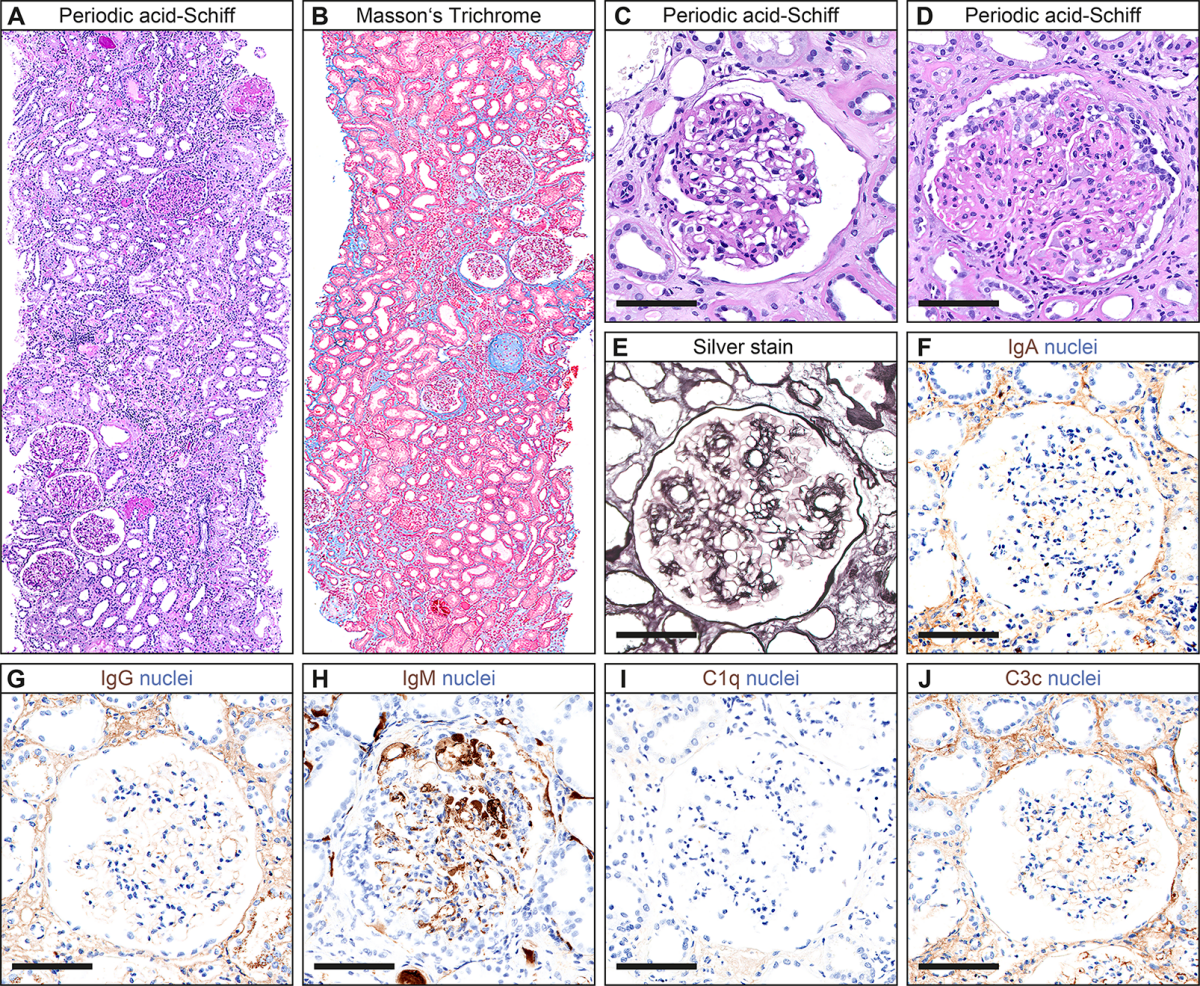
Histopathological findings. **(A, B)** Representative kidney sections stained with periodic acid-Schiff and Masson’s Trichrome showing acute tubular injury and mild interstitial fibrosis. **(C)** Glomeruli showed mild mesangial expansion according to Tervaert class IIa. **(D)** Notably, collapsing FSGS was present. **(E)** As shown by silver stain, no double contours of the basement membrane were detectable. **(F–J)** No subendothelial immune deposits (IgA, IgG, IgM, C1q, and C3c) were detectable, while IgM was entrapped in damaged glomerular capillaries. Scale bars: 100 μm. Abbreviations: C1q, complement component 1q; C3c, complement factor 3 conversion product; FSGS, focal segmental glomerulosclerosis; IgA, immunoglobulin A; IgG, immunoglobulin G.

A 60-year-old Caucasian woman had known arterial hypertension, insulin-dependent type 2 diabetes, and chronic kidney disease (CKD) stage 1. A left axillary abscess incision identified incident breast invasive ductal carcinoma, grade 3 with positivity for estrogen receptor (ER; 80%), progesterone receptor (PR; 80%), Ki-67 (60%), and HER2. Based on diagnosis of metastatic breast cancer and reduced physical condition, the patient received neoadjuvant therapy with paclitaxel (80 mg/m^2^ weekly), pertuzumab (loading dose 840 mg, 420 mg every 3 weeks thereafter), and trastuzumab (loading dose 8 mg/kg body weight, 6 mg/kg every 3 weeks thereafter) because of HER2 positivity for 2 months. Because of coronavirus disease 2019 (COVID-19) requiring hospitalization for 1 week and delayed convalescence, the chemotherapy was paused after three cycles of paclitaxel and two cycles of pertuzumab/trastuzumab and re-initiated thereafter. After a total number of 12 cycles of paclitaxel and seven cycles of pertuzumab/trastuzumab, left axillary lymph node dissection (ALND) was performed (66/102 metastatic lymph nodes; ER, 100%; PR, 90%; Ki-67, 60%; HER2, heterogeneous positive), and adjuvant T-DM1 chemotherapy (3.6 mg/kg body weight every 3 weeks, 302 mg) was initiated thereafter. Four weeks prior to admission, the patient received the first infusion with T-DM1 followed by a second infusion 1 week prior to admission (**Figure 1**). At admission, the patient presented to our emergency department with hypertensive crisis (initial blood pressure of 211/126 mmHg), acute kidney injury (AKI) stage 1, proteinuria (4.4 g/g creatinine), and albuminuria (3.1 g/g creatinine, normal range <30 mg/g, **Figure 1** and **Table 1**). A repeat nasopharyngeal swab for SARS-CoV-2 RNA testing by PCR at admission was negative. Laboratory tests excluded tumor lysis syndrome and rhabdomyolysis; serological evaluation was normal for antinuclear antibodies (ANA), anti-neutrophil cytoplasmic antibodies (ANCA), and anti-glomerular basement membrane (GBM); and no reduction in serum complement C3 and C4 was observed (**Table 1**). During the further course, the patient presented with deterioration of kidney function (serum creatinine up to 2.25 mg/dl, normal range 0.5–1 mg/dl, **Figure 1**), in addition to progressive nephrotic range proteinuria (up to >10 g/g creatinine), albuminuria (up to 6.7 g/g creatinine, normal range <30 mg/g), and hematuria. A kidney biopsy was performed showing acute tubular injury and mild interstitial fibrosis (**Figures 2A, B**). Glomeruli showed moderate mesangial expansion according to Tervaert class IIb (**Figure 2C**) (7). Notably, collapsing FSGS was present (**Figure 2D**). Severe vascular hyalinosis as previously described specifically in collapsing FSGS superimposed in diabetic nephropathy was not present (8). Moreover, no double contours of the GBM were detectable, thereby excluding collapsing FSGS due to chemotherapy-associated thrombotic microangiopathy (TMA; **Figure 2E**) (9). Furthermore, no subendothelial immune deposits (IgA, IgG, IgM, C1q, and C3c) were detectable, while IgM was entrapped in damaged glomerular capillaries (**Figures 2F–J**). After discontinuation of T-DM1 therapy, proteinuria (down to 1.1 g/g creatinine) and albuminuria (down to 0.7 g/g creatinine) improved 3 weeks after the last T-DM1 infusion without further specific treatment, associated with improvement of kidney function (serum creatinine 1.13 mg/dl, normal range 0.5–1 mg/dl, **Figure 1**).

**Table 1:** Key clinical parameters at presentation.

Parameter	Value	Normal range
Serum creatinine, mg/dl	1.49	0.7–1.2
eGFR, ml/min/1.73 m^2^	37.9	>60
BUN, mg/dl	22	8–26
CRP, mg/L	15.2	≤5.0
LDH, U/L	263	125–250
CK, U/L	92	29–168
Anti-GBM, U/ml	<0.8	<7
ANA IF	1:100	<1:100
ANCA IF	Neg	Neg
C3c, g/L	1.78	0.82–1.93
C4, g/L	0.58	0.15–0.57
uPCR, mg/g	4,381	<300
uACR, mg/g	3,069	<30
Urinary kappa, mg/L	257	<6.8
Urinary lambda, mg/L	153	<3.7
Urinary kappa/lambda, ratio	1.68	>1 or <5.2

ANA, antinuclear antibodies; ANCA, anti-neutrophil cytoplasmic antibodies; C3c, complement factor 3 conversion product; C4, complement factor 4; CRP, C-reactive protein; eGFR, estimated glomerular filtration rate (CKD-EPI); GBM, glomerular basement membrane; Neg, negative; uACR, urinary albumin-to-creatinine ratio; uPCR, urinary protein-to-creatinine ratio; BUN, blood urea nitrogen; LDH, lactate dehydrogenase; CK, creatine kinase.

## Discussion

An analysis of the FDA Adverse Event Reporting System identified 124/1,243 (10%) renal adverse events (defined as proteinuria, AKI, elevated serum creatinine, and/or nephritis) for trastuzumab, reported between 2011 and 2015 (6). However, there are no published reports describing kidney biopsy findings related to nephrotoxicity of either trastuzumab or T-DM1. To our knowledge, we here provide the first report of kidney biopsy findings in a case of nephrotic range proteinuria due to collapsing FSGS after initiation of T-DM1 therapy. Collapsing glomerulopathy is a pattern of glomerular injury characterized by shriveling of the glomerular tuft in the setting of FSGS (10). Primary variants are most often observed in subjects of African ancestry with a genetic predisposition who carry an *APOL1* high-risk genotype (homozygous for G1 or G2 or compound G1/G2 heterozygotes) and triggering diseases that behave like a “second hit” leading to clinical manifestation of collapsing FSGS (11). Collapsing FSGS has also been described secondary to infection (including HIV and parvovirus), drugs (bisphosphonate and calcineurin inhibitors), severe vascular disease (TMA and cocaine use), and autoimmune diseases like systemic lupus erythematosus (SLE) (11–19). However, these conditions were excluded in our patient. Together with the fact that the kidney biopsy showed only moderate mesangial expansion (according to Tervaert class IIb) without advanced vascular hyalinosis previously described specifically in collapsing FSGS superimposed in diabetic nephropathy, it is likely that the observed collapsing FSGS was associated with the prior initiation of T-DM1 therapy (8). We also observed acute tubular injury often observed in kidney biopsies of patients presenting with nephrotic range proteinuria (20). Collapsing FSGS in association with T-DM1 is further supported by the clinical course with improvement of proteinuria and kidney function 3 weeks after discontinuation of T-DM1 therapy without further specific treatment. Since T-DM1 is an antibody–drug conjugate consisting of the monoclonal antibody trastuzumab linked to the maytansinoid DM1, we cannot conclude that observed collapsing FSGS is attributed to either drug conjugate or both.

## Conclusions

In summary, we report kidney biopsy findings in a case of nephrotic range proteinuria due to collapsing FSGS and tubular injury after initiation of T-DM1 therapy. This issue is especially relevant since T-DM1 is widely used and nephrologists have to be aware of this potentially rare but severe complication.

## Data Availability Statement

The original contributions presented in the study are included in the article/supplementary material. Further inquiries can be directed to the corresponding author.

## Ethics Statement

Ethical review and approval was not required for the study on human participants in accordance with the local legislation and institutional requirements. The patients/participants provided their written informed consent to participate in this study.

## Author Contributions

SH and BT conceived the study, collected and analyzed the data, and co-wrote the first draft. SH evaluated the histopathological findings. SW and JG edited the manuscript. SW, JG, and BT were directly involved in the treatment of the patient. All authors contributed to the article and approved the submitted version.

## Funding

We acknowledge support from the Open Access Publication Funds of the Georg August University Göttingen.

## Conflict of Interest

The authors declare that the research was conducted in the absence of any commercial or financial relationships that could be construed as a potential conflict of interest.

## Publisher’s Note

All claims expressed in this article are solely those of the authors and do not necessarily represent those of their affiliated organizations, or those of the publisher, the editors and the reviewers. Any product that may be evaluated in this article, or claim that may be made by its manufacturer, is not guaranteed or endorsed by the publisher.
